# Antiproliferative, Antiangiogenic, and Antimetastatic Therapy Response by Mangiferin in a Syngeneic Immunocompetent Colorectal Cancer Mouse Model Involves Changes in Mitochondrial Energy Metabolism

**DOI:** 10.3389/fphar.2021.670167

**Published:** 2021-12-03

**Authors:** Julio César Rodriguez-Gonzalez, Ivones Hernández-Balmaseda, Ken Declerck, Claudina Pérez-Novo, Emilie Logie, Claudia Theys, Patrycja Jakubek, Olga Luisa Quiñones-Maza, Geovanni Dantas-Cassali, Diego Carlos dos Reis, Guy Van Camp, Miriam Teresa Lopes Paz, Idania Rodeiro-Guerra, René Delgado-Hernández, Wim Vanden Berghe

**Affiliations:** ^1^ Centro de Investigación y Desarrollo de Medicamentos (CIDEM), La Habana, Cuba; ^2^ Laboratorio de Farmacología, Instituto de Ciencias del Mar (ICIMAR), CITMA, La Habana, Cuba; ^3^ Laboratory of Protein Science, Proteomics and Epigenetic Signaling (PPES) and Integrated Personalized and Precision Oncology Network (IPPON), Department of Biomedical Sciences, University of Antwerp, Campus Drie Eiken, Antwerp, Belgium; ^4^ Department of Food Chemistry, Technology and Biotechnology, Faculty of Chemistry, Gdansk University of Technology, Gdansk, Poland; ^5^ Departamento de Farmacología, Instituto de Ciencias Biológicas (ICB), Universidad Federal de Minas Gerais (UFMG), Horizonte, Brazil; ^6^ Center of Medical Genetics, University of Antwerp and Antwerp University Hospital, Antwerp, Belgium; ^7^ Centro de Estudios para las Investigaciones y Evaluaciones Biológicas (CEIEB), Instituto de Farmacia y Alimentos (IFAL), Universidad de La Habana, La Habana, Cuba; ^8^ Facultad de Ciencias Naturales y Agropecuarias, Universidat de Santander (UDES), Bucaramanga, Colombia

**Keywords:** Mangiferin (PubChem CID: 5081647), antitumor, antiangiogenesis, antimetastasic, colon carcinoma, mitochondrial metabolism

## Abstract

In spite of the current advances and achievements in cancer treatments, colorectal cancer (CRC) persists as one of the most prevalent and deadly tumor types in both men and women worldwide. Drug resistance, adverse side effects and high rate of angiogenesis, metastasis and tumor relapse remain one of the greatest challenges in long-term management of CRC and urges need for new leads of anticancer drugs. We demonstrate that CRC treatment with the phytopharmaceutical mangiferin (MGF), a glucosylxanthone present in Mango tree stem bark and leaves (*Mangifera Indica L.*), induces dose-dependent tumor regression and decreases lung metastasis in a syngeneic immunocompetent allograft mouse model of murine CT26 colon carcinoma, which increases overall survival of mice. Antimetastatic and antiangiogenic MGF effects could be further validated in a wound healing *in vitro* model in human HT29 cells and in a matrigel plug implant mouse model. Interestingly, transcriptome pathway enrichment analysis demonstrates that MGF inhibits tumor growth, metastasis and angiogenesis by multi-targeting of mitochondrial oxidoreductase and fatty acid β-oxidation metabolism, PPAR, SIRT, NFκB, Stat3, HIF, Wnt and GP6 signaling pathways. MGF effects on fatty acid β-oxidation metabolism and carnitine palmitoyltransferase 1 (CPT1) protein expression could be further confirmed *in vitro* in human HT29 colon cells. In conclusion, antitumor, antiangiogenic and antimetastatic effects of MGF treatment hold promise to reduce adverse toxicity and to mitigate therapeutic outcome of colorectal cancer treatment by targeting mitochondrial energy metabolism in the tumor microenvironment.

## Introduction

The latest GLOBOCAN 2018 database showed that colorectal cancer (CRC) is placed third in incidence and second in mortality worldwide, respectively; being responsible for 10% of all newly diagnosed cancer cases, and 9% of all cancer deaths worldwide (Bray et al., 2018). In Cuba, CRC ranked fifth in incidence and third in mortality in 2013, being more frequently in female and in adult people over 60 years of age in both indicators (Ministerio de Salud Pública.Dirección de Registros Médicos y Estadísticas de Salud and de Cuba, 2017). Despite advances in the prevention and diagnosis of CRC, between 60 and 70% of patients are detected at middle- or late-stage CRC, which results in difficult clinical management and less favourable prognosis (Petrelli et al., 2017). This contributes to 40–50% mortality rate within the first 5 years since the diagnosis, accounting for almost 10% of the cancer mortalities each year (Torre et al., 2016). Most of the CRC patients die from metastases (mCRC), predominantly in the liver, lungs, lymph nodes, and the peritoneum (Vatandoust et al., 2015). The main therapeutic options to mCRC are the surgical removal of liver metastases and/or chemotherapy. The former has increased patient survival in the first 5 years, but only 20–25% of these patients are eligible for surgery (Dhir and Sasson, 2016). The effectiveness of current chemotherapies is limited (Pritchard et al., 2012) due to suppression of host immune antitumor responses (Teng et al., 2015; Bhatia and Kumar, 2016), therapy-induced tumor resistance, tumor relapse (Shaked, 2016) local/systemic toxicities and increased risk of secondary tumorigenesis (Pritchard et al., 2012; Abdullah and Chow, 2013). In this scenario more than 80% of patients with mCRC die during the long-term follow-up (Ting et al., 2013). The progression of CRC is also closely related to angiogenesis associated with a poor prognosis and relapse of the disease (Mihalache and Rogoveanu, 2014) and has become an alternative valid therapeutic target in the treatment of colon cancer (Wang et al., 2015; Wang and Zhu, 2016). Since 2004, conventional chemotherapy is combined with targeted monoclonal antibody therapies specifically directed against vascular endothelial growth factor (VEGF) and epidermal growth factor (EGF) and their receptors to block angiogenic and proliferative tumor actions (Mihalache and Rogoveanu, 2014), (Mayer, 2009). Unfortunatedly, resistance and adverse effects have also been reported for these targeted treatments (Wang et al., 2015; Wang and Zhu, 2016). This has renewed interest in phytochemicals from medicinal plants for CRC treatment. Herbs with a rich ethnomedicinal history represent a rich resource of bioactive compounds with polypharmaceutical effects with multiple targets in cell growth, cell differentiation, and apoptosis regulation (Chen et al., 2008; Newman and Cragg, 2020; Atanasov et al., 2021). The public WHO global report on traditional and complementary medicine indicates that in developing countries, 80% of population relies on plant-derived medicines for the health care (World Health Organization, 2019). The number of phytochemical products submitted to the FDA is particularly high in the oncological area.

Mangiferin (MGF) is a polyphenolic phytochemical of the C-glycosylated xanthone type (1,3,6,7-tetrahydroxyxanthone-C2-β-d glucoside) present in all parts of *Mangifera indica* L. (*Anacardiaceae*), and in some medicinal plants. We and others have previously demonstrated promising antiproliferative, anti-angiogenic, anti-invasive and antimetastatic antitumor effects by poorly characterized mechanisms in different cancer cell types ((Leiro et al., 2004; Sarkar et al., 2004; García-Rivera et al., 2011; Khurana et al., 2016; Núñez Selles et al., 2016; Imran et al., 2017; Zou et al., 2017; Tan et al., 2018; He et al., 2019; Delgado-Hernández et al., 2020)).

Taking into account the interesting pharmacological properties of MGF described above, we applied a systems biology transcriptomics approach to resolve the molecular mechanism of action for the *in vivo* antitumor effects of MGF in a syngeneic immunocompetent allograft mouse model of murine CT26 colon carcinoma.

## Materials and Methods

### Reagents

Mangiferin (MGF) was purified and characterized from the aqueous extract of the stem bark and leaves of *Mangifera indica* L to 95,5% purity at the Departments of Pharmacognosy, Institute of Food and Pharmacy, Havana University and Department of Analytical Chemistry of Center of Drugs Research and Development, CIDEM (Núñez Sellés et al., 2002; García-Rivera et al., 2011; Gil et al., 2014). Cisplatin (cis-diamminedichloroplatinum(II), CDDP) was purchased from Shandong Boyuan Pharmaceutical Co., Ltd, China. The culture medium RPMI-1640, dimethylsulfoxide (DMSO), fetal bovine serum, penicillin-streptomycin (100x), L-Glutamine, trypsin, ethylenediaminetetraacetic acid (EDTA), sodium dodecyl sulphate (SDS), reduced Matrigel (196 USP unit/ mg) and set of Drabkin reagents were purchased from Sigma-Aldrich Inc. (Saint Louis, MO, United States). 3-(4,5-Dimethyl-2-thiazolyl)-2,5-diphenyl-2H-tetrazolium bromide (MTT), 2-propanol, ethanol, hydrochloric acid (HCl), formaldehyde and acetic acid were obtained from Merck KGaA (Germany). Solutions of test compounds were routinely prepared in DMSO or MilliQ water and sterilized using syringe-driven 0.22 µm filters. The final concentration of DMSO in culture medium did never exceed 0.5%.

### Tumor Cell Lines and Culture Conditions

CT26. WT is an *N*-nitroso-*N*-methylurethane-(NNMU) induced undifferentiated murine adenocarcinoma of the colon, syngeneic with the BALB/c mouse, which was provided by the Molecular Immunology Center, Havana. CT26. WT cells were grown in cell culture as monolayers in T-75 cm^2^ culture flasks (BD Falcon) in RPMI-1640 with 2 mM of L-Glutamine supplemented with 10% fetal bovine serum, 100 UI/mL of penicillin, and 100 μg/ ml of streptomycin. Only cells of the first two serial passages after cryostorage were used in the syngeneic allograft models (Evans et al., 2016). At the day of implantation, the cells were harvested from subconfluent cultures (70–85%) by trypsinization (0.05% trypsin and 0.02% EDTA), and washed twice in phosphate-buffered saline solution before they were resuspended in non-supplemented RPMI culture medium. Human colon adenocarcinoma cell line HT29 (ATCC HTB-38) was cultured in McCoy’s 5 A medium (Gibco, ThermoFisher, United States) supplemented with 1.5 mM L-glutamine, 2.2 g/L sodium bicarbonate, 100 ml/ L of fetal bovine serum and 100 U/ mL penicillin and 100 g/ L streptomycin. All cell lines were cultured in a humidified atmosphere (37°C, 5% CO_2_).

### Cell Viability Assays

MTT assays were performed to assess cell viability and/or growth inhibition of CT26 and HT29 colon cancer cell lines after exposure to MGF essentially, as previously described (Hernandez-Balmaseda et al., 2021). Briefly, cells were seeded in 96-well tissue culture plates at the concentration of 5,000 cells per well. The next day cells were treated with MGF in a concentration range of 10–400 µM for 24, 48, and 72 h. Control cells were exposed to 0.5% DMSO solvent control. Following treatment, the medium supernatant was aspirated from the wells and replaced with fresh medium. Next, 50 µL of MTT solution was added to all the wells and cells were incubated for another 4 h at 37°C. Subsequently, medium was aspirated and formazan crystals were dissolved in 50 µL of DMSO. The measurement of absorption was performed at 595 nm using (Envision 2103 multilabel reader, Perkin Elmer, United States) plate reader. The experiments were repeated with four technical replicates per treatment.

### Cell Migration Wound Scratch Assay

HT29 cells were seeded at a concentration of 35,000 cells per well and left to grow at 37°C under 5% CO_2_ until they reached 95–100% confluence. Then, cells were treated with 10 μg/ ml of MMC for 4 h to block cell proliferation and distinguish it from cell migration. When the incubation time was finished, a scratch was made in each well with the Incucyte Woundmaker tool. Next, cells were gently washed with PBS to remove any cell debris formed after making a scratch. Afterwards PBS was removed and exchanged with serum-free McCoy’s 5 A medium to prevent cell division. The cells were treated either with MGF alone (at 1, 50 and 400 µM concentrations) or in combination with a proangiogenic cell migration growth factor VEGF (at 10 ng/ ml) for 24 h and incubated at 37°C under 5% CO_2_ in the Incucyte Live-Cell Analysis system (Sartorius, Germany). Control cells were treated with an appropriate solvent, which in the case of MGF was 0.5% DMSO and in the case of VEGF–MilliQ water. Imaging was performed every 2 h for a total duration of 24 h with a × 10 objective. The area of wound closure was calculated and analysed using the Incucyte Scratch Wound Cell Migration software module of the ZOOM imaging system. The experiments were performed in three biological replicates with three technical replicates for each treatment.

### Fatty Acid β-oxidation and Ketone Body Assays

Intracellular lipid accumulation induced by free fatty acid exposure leads to enhanced fatty acid oxidation (FAO) and ketogenesis (beta-hydroxybutyrate production). Protein expression levels of CPT1 (carnitine palmitoyltransferase 1), a key regulatory enzyme of mitochondrial fatty acid β-oxidation were determined by Western analysis. Ketone body (beta-hydroxybutyrate) levels were assessed using a colorimetric assay kit (Cayman Chem). Briefly HT29 cells were seeded at a density of 18*10^6^ cells in a T175 flask. The next day cells were treated with 0.2 mM FFA and 40 µM or 400 µM MGF for 24 h. Stock solutions of 0.66 M oleic acid and (1.32 M) palmitic acid (Sigma-Aldrich, Germany) were prepared in isopropanol. Equal amounts of oleic and palmitic acid were mixed to prepare a FFA stock of 1 mM FFA. Free fatty acid-free bovine serum albumin (BSA) was dissolved in serum-free McCoy’s 5 A or RPMI medium without antibiotics at the final concentration of 1% and then sterilized using syringe-driven 0.22 µm filters. Afterwards, medium was supplemented with the mixture of FFA at a final concentration of 0.2 mM and sonicated for 6–8 h until FFA were completely dissolved using a Branson 3,200 sonication bath. FFA medium was protected from light and stored at 4°C. Following treatment, cell pellets were collected with a cell scraper and used immediately to extract ketone bodies according to the manufacturers protocol. The measurement of absorption was performed at 450 nm using (Envision 2103 multilabel reader, Perkin Elmer, United States) plate reader at room temperature. The experiments were performed in one biological replicate, with three technical replicates per each treatment. Similarly, RNA was extracted using RNeasy Mini Kit (Qiagen, Germany) from the collected cell pellets. RNA quantity was determined using Qubit™ RNA Broad Range Assay Kit with the aid of the Invitrogen Qubit™ 4 Fluorometer (Thermo Fisher Scientific, United States). The extracted RNA was stored at -80°C until further QPCR analysis. For protein analysis, cell pellet was washed once with cold PBS, centrifuged (1,500 rpm, 3 min, 4°C) then cells were lysed using 1x RIPA buffer (Cell Signalling, United States) with a cocktail of protease inhibitors (Sigma-Aldrich, Germany) and left on ice for 15 min. Afterwards, cell lysates were centrifuged (13,000 rpm, 15 min, 4°C) and the supernatants were transferred to the new test tubes. Protein concentration was measured using Pierce™ BCA Protein Assay Kit (Thermo Fisher Scientific, United States) and the measurement of absorption was performed at 560 nm using (Envision 2103 multilabel reader, Perkin Elmer, United States) plate reader. Prior to SDS-PAGE electrophoresis, protein lysates were mixed with Laemmli buffer (Biorad, United States) and 50 mM 1,4-dithiothreitol (DTT) and then heated at 70°C for 10 min. Then, the samples and protein ladder (BenchMark™ Protein Ladder, Thermo Fisher Scientific, United States) were loaded on SDS-PAGE polyacrylamide gels (stacking 6%, resolving 12%) at protein concentration equal to 10 µg/well. The electrophoresis was run in Mini-PROTEAN Tetra Cell System (Biorad, United States) using the high molecular weight buffer (100 mM MOPS, 100 mM Tris, 0.2% SDS, 2 mM EDTA, 5 mM sodium bisulfite) at 70 V until the samples reached the separating gel and then the voltage was increased up to 120 V. Afterwards, the proteins were transferred on pre-wet nitrocellulose membranes (Cytiva, United States) in Power Blotter 1-step transfer buffer (Invitrogen, United States) using Power Blotter XL System (Invitrogen, United States) (7 min at 1.3 A). Nitrocellulose membranes were blocked in blocking buffer (5% milk solution in TBST) for 1 h at room temperature and incubated overnight at 4°C with primary antibodies diluted in blocking buffer as follows: 1:4000 for anti-CPT1A (Proteintech, UK). Next day, membranes were washed three times with TBST and then incubated with HRP-conjugated anti-rabbit secondary antibody diluted in blocking buffer (1:2000). Anti-GAPDH (Cell signaling, United States) antibodies (diluted 1:2000) in blocking buffer were used as loading controls for CPT1A. Protein detection was performed on the Amershan imager 680 (Cytiva, United States) using Pierce™ ECL Western Blotting Substrate (Thermo Fisher Scientific, United States) and quantified using Image-J software.

### *In vivo* Experiments

Housing and all procedures involving animals were performed according to protocols approved by the Drug Research and Development Center Ethics Committee for Animal Research and in compliance with the Cuban National Guidelines for the Care and Use of Laboratory Animals (CECMED Resolution No. 37 2012 for Good Laboratory Practice for Quality Control). Male BALB/c mice were purchased at National Laboratory Animal Production Center, Havana, Cuba. BALB/c mice were used at 6–8 weeks of age with weight between 18 and 22 g, in all experiments. They were supplied with water and food at *libitum* and were kept in quarantine for 15 days for adaptation to temperature (22°C ± 2°C), humidity (77 ± 3%) and alternating cycles of 12 h light/dark environment. For allograft studies, the animals were inoculated (as specified in more detail for each specific model below) with 1x10^5^ CT26. WT cells/mouse in 100 μL of non-supplemented RPMI-1640 culture medium. The following day, they were weighted, labeled and placed in separate boxes.

#### Ectopic Subcutaneous Syngeneic CT26. WT Tumor Model

The animals were inoculated subcutaneously with CT26. WT cells on the right dorsal side. Once tumors grew up to 30 mm^3^, mice were randomly divided at day 14 into five groups (10 animals/group) and treated for 14 days (28 days protocol). The products, doses, and treatment schedules were as follows: *Group I*, carboxymethylcellulose (CMC) 0.05% as the negative control; *Group II*, MGF 10 mg/ kg b. w.; *Group III*, MGF 50 mg/ kg b. w.; *Group IV*, MGF 100 mg/ kg b. w.; and *Group V*, CDDP 5 mg/kg b. w. as positive control. MGF doses were prepared in 0.05% CMC and CDDP in sterile saline solution (0,9% NaCl). MGF and CMC were administered daily intragastrically and CDDP every 6 days by intraperitoneal injections in three treatment cycles. The tumor growth was monitored and tumor size was measured by length and width with caliper every 2 days by the same operator, and the tumor volume was calculated (V, mm^3^ = length x width^2^ x 0.5). At the end of the experiment, all mice were sacrificed and dissected. The tumors were recovered for photographic imaging and weighed. Three independent experiments were performed.

#### Lung Metastasis Model

The animals were challenged by an intravenous puncture in the tail veins and distributed in five groups (10/group). The products, doses, and treatment regimens were as follows: *Group I*, 0.05% CMC; *Group II*, MGF 10 mg/kg b. w.; *Group III*, MGF 50 mg/ kg b. w.; *Group IV*: MGF 100 mg/ kg b. w.; and *Group V*, CDDP 5 mg/kg b. w. The preparation, route, and frequency of administration were the same as those described in the ectopic/subcutaneous model. The animals were treated for 17 days and sacrificed at day 18. The trachea-lung were extracted, photographed and perfused with 20 Gx1½ tracheal needles with 1 ml 15% Chinese ink in PBS pH 7.2–7.4. The trachea was excised and the lungs were soaked for discoloration and fixation for 5 min in Fekete’s solution (Bao et al., 2011). The lungs were photographed and the superficial metastatic nodules were counted using a stereoscope (Olympus, Japan) and fixed with 4% formaldehyde. Lungs presented with more than 250 nodules, were assigned a cut-off value of 250 (due to the difficulty of counting a larger number because of high nodule density). A survival analysis was performed until the treatments were completed. Three independent experiments were performed.

#### Angiogenesis Model by Matrigel Implant

The animals were divided into four groups (5/group). The control groups were implanted with 200 μL of a cold mixture of Matrigel, heparin (32 IU) and PBS (*negative or basal control: implant A*) and another mixture equal to the previous one plus CT26. WT cells (5 x 10^5^) as *positive control: implant B*. For the treatment groups, two concentration of MGF: *2*00 μg/ ml (*implant C*) and 100 μg/ ml(implant D) were included into the implant volume B. The implants were subcutaneously applied in the in the inguinal region. The experimentation time was extended by 12 days. The animals were sacrificed, the plugs were excised, photographed and cleaved with residues of surrounding tissue to facilitate histological orientation. They were weighed and measured with caliper by their length, width, and height, and the volume was calculated using the formula of an ellipsoid: V (mm^3^) = length x width x height x π/6). The plugs were liquified in 300 μL PBS by incubation at 4°C, overnight (Beatty and Paterson, 2001), and the hemoglobin (Hb) released was quantified by the Drabkin method (Drabkin and Austin, 1935). For histological analysis, the plugs were fixed in 10% formalin in PBS, embedded in paraffin and cut (5–7 μm). Histological staining was initially performed with hematoxylin-eosin and whereas specific staining for blood vessel counts was done with Masson’s trichrome. A microscopic count of the blood vessels was performed throughout the periphery of the tumor.

### Immunohistochemical Studies

Subcutaneous tissue tumors were fixed in formalin (10% w/v in phosphate-buffered saline–PBS pH 7.4) during 24 h and sections (4 µm) were processed for immunohistochemistry analysis. A biotin-peroxidase system was used with identification of the secondary antibody by polymer (ADVANCE HRP). Antigen retrieval was performed in water bath at 98°C, with a citrate buffer solution (pH 6.0) (Target Retrieval Solution) for 20 min. In order to block the endogenous peroxidase activity, slides were incubated with a solution of H_2_O_2_ 3% in methyl alcohol. Reagents were applied manually with 1 h incubation for the primary antibody and 30 min for the reagents, except for chromogen diaminobenzidine, incubated for 5 min. Minichromosome maintenance complex component 7 (MCM 7 or CDC47), a marker of cell proliferation, was measured using a specific monoclonal antibody CDC47 (clone 47DC141, Neomarkers, 1/300) (Zorde Khvalevsky et al., 2013). Positive and negative controls were included in each bath. The index for CDC47 staining was obtained by counting the percentage of positive cells in 500 tumor cells.

### RNA Extraction and Isolation

Subcutaneous and pulmonary tumor tissue samples from each animal (n = 5/group) were individually treated with RNA*later* ^®^ (Qiagen, Benelux, Belgium) by 24 h at 4°C, and immediately stored at −80°C until the RNA isolation procedure. Up to 30 mg tissue were completely disrupted and homogenized in 700 μL QIAzol^®^ Lysis Reagent (Qiagen, Benelux, Belgium) by the Tissue Lyser II (Qiagen^®^, Benelux, Belgium) with 5 mm stainless steel beads diameter to extract total RNA. Total RNA was isolated using RNeasy^®^ Microarray Tissue kit (Qiagen, Benelux, Belgium), according to manufacturer’s protocol. Total RNA was purified on RNeasy Mini spin columns according to the manufacturer’s protocol. During the purification process contaminating genomic DNA was removed by means of DNase digestion. Purity and concentration total RNA samples were evaluated by ultraviolet spectroscopy (NanoDrop, Thermo Scientific, Wilmington, United States). The integrity of the RNA samples was evaluated by the Experion Automated Electrophoresis System (BioRad, CA, United States) according to the manufacturer instructions. Good quality of the RNA material samples with A260/280 ratio between 1,9–2,1 were used for further analysis.

### RNA Bead Array and Data Processing

Five hundred ng of total RNA from 18 samples (3 replicates by group) subcutaneous and pulmonary tissue tumors were amplified using the Illumina TotalPrep RNA Amplification kit (Life Technologies, Carlsbad, CA, United States). RNA was reverse transcribed using T7 oligo(dT) primers after which biotinylated cRNA was synthesized through *in vitro* transcription reaction. Seven hundred 50 ng of amplified cRNA was hybridized to a corresponding array on a Mouse WG-6 2v bead chip (Illumina, San Diego, CA, United States). The beadchip array was incubated for 18 h at 58°C in a hybridization oven whilst continuous rocking. After several consecutive washing steps, bead intensities were read on Illumina iScan. Raw data intensities were read in R using the “bead array” package (v2.8.1) (Dunning et al., 2007). Intensities were quantile normalized and differential gene expression between samples was estimated using “limma” (v3.14.1) (Ritchie et al., 2015). Genes were considered to be differentially expressed when they had a p-value < 0.05 and a fold change of at least 1.25 in comparison to controls. Transcriptomic analysis was done by uploading Illumina bead array gene expression data into Ingenuity Pathway Analysis (IPA) software (Ingenuity^®^ Systems, www.ingenuity.com, Redwood City, CA, United States and QIAGEN Inc., https://digitalinsights.qiagen.com/products-overview/discovery-insights-portfolio/analysis-and-visualization/qiagen-ipa/?), and performing a core analysis according to the instructions provided. Each identifier was mapped to its corresponding gene object in the Ingenuity knowledge base. Fisher’s exact test was used to calculate a p-value determining the probability that each biological function and/or disease assigned to that data set is due to chance alone. Metascape systems biology freeware (https://metascape.org/) was used for correlating the transcriptomic profiles of the different *in vivo* data (Zhou et al., 2019). The Circos plot visualization allows to show how genes from different input gene lists overlap. Heatmaps show Metascape enrichment analysis of all statistically enriched ontology terms (GO/KEGG terms, canonical pathways, hall mark gene sets). Accumulative hypergeometric p-values and enrichment factors are calculated and used for filtering. Remaining significant terms are then hierarchically clustered into a tree dendrogram based on Kappa-statistical similarities among their gene memberships. The term with the best p-value are selected within each cluster as a representative term to be displayed in a hierarchical tree dendrogram. The heatmap cells are colored by their p-values (see color legend). Along the same line, Metascape enrichment analysis of all statistically enriched transcription factor (TF)-target interaction networks is dermined by the TRRUST database (Han et al., 2018). Protein-protein interactions (PPI) among all input gene lists are extracted from PPI data source to form a PPI network. GO enrichment analysis is applied to the network to assign biological “meanings” of sub-protein networks. GO enrichment analysis is applied to each MCODE network to assign “meanings” to the network component, where top three best p-value terms were retained. MCODE components were identified from the merged network. Each MCODE network is assigned a unique color.

### Reverse Transcription-PCR and Real-Time Quantitative PCR Validation

Total RNA (700 ng) isolated by RNeasy Mini Kit (Qiagen), was reverse transcribed to cDNA by the GoScript™ Reverse Transcriptase kit (Promega, United States) according to the manufacturer’s protocol. RT-qPCR was performed using GoTaq^®^ qPCR master mix (Promega, United States) on Rotor-Gene Q (Qiagen, Germany). Gene expression was calculated with the ΔΔCt method after normalization against the glyceraldehyde-3-phosphate dehydrogenase (*GAPDH*), actinB (*ACTB*) or beta-2-microtubulin (*B2M*) housekeeping genes, as further specified. Following gene specific primersets were used for array validation experiments of C-C Motif Chemokine Ligand 3 (*CCL3*), Collagen Type VI Alpha three chain (*COL6A3*), C-X-C Motif Chemokine Ligand 9 (*CXCL9*) and Metalloproteinase 13 (*MMP13*); and the following gene specific primer sets: *CCL3* forward primer: 5′-CAG​CCA​GGT​GTC​ATT​TTC​CTG-3′ and reverse primer: 5′-AGG​TCT​CTT​TGG​AGT​CAG​CG-3´. *COL6A3* forward primer: 5′-GGA​ACC​ACG​GAA​GAG​AGC​AA-3′ and reverse primer: 5′-CAGGGAACTGACCCAAGACA-3´.*CXCL9* forward primer: 5′- TGT​GGA​GTT​CGA​GGA​ACC​CT-3′ and reverse primer: 5′- AGT​CCG​GAT​CTA​GGC​AGG​TT-3´. *MMP13* forward primer: 5′- GGA​GCC​CTG​ATG​TTT​CCC​AT-3′ and reverse primer: 5′- GTC​TTC​ATC​GCC​TGG​ACC​ATA-3´. The experiment was carried out in duplicate in three independent biological replicates. The thermal cycling conditions included initial denaturation at 95°C for 2 min and subsequent 40 cycles of two-step protocol: denaturation at 95°C for 15 s and annealing/extension at 60°C for 60 s.

### Statistical Analysis

One-way or two-way ANOVA with Dunnett post hoc test (comparison every mean to control mean) were performed to evaluate statistical significance, as indicated in Figure legends. Survival analysis was done with the log-rank (Mantel-Cox) statistical test. All statistical analyses were performed using GraphPad Prism^®^ (GraphPad Software, La Jolla California, United States) statistical package. P value < 0.05 was considered significant.

## Results

### Mangiferin Induces CT26.WT Colorectal Tumor Regression *in vivo*

**FIGURE 1:**
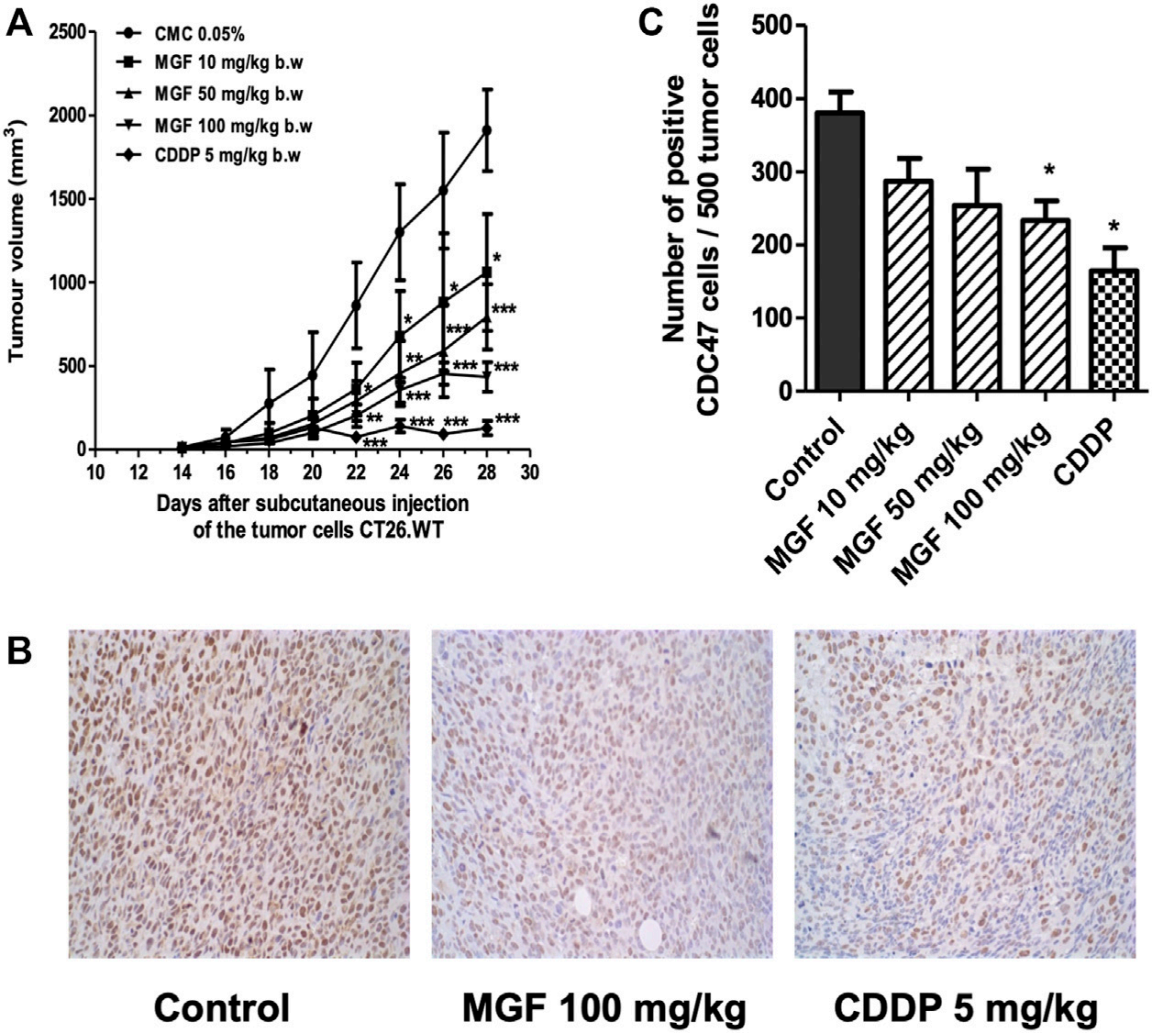
MGF treatment induces tumor regression in CT26. WT colorectal cancer model. BALB/c mouse colorectal cancer cells CT26. WT (1 × 10^5^ cells) were injected subcutaneously into 6–8 weeks-old BALB/c mice. After 14 days of tumor allograft growth, the mice were daily treated for 14 days by (oral) cannulation with MGF at low (10 mg/ kg), intermediate (50 mg/ kg) and high (100 mg/ kg) doses or carboxymethyl cellulose (CMC) 0,05% as vehicle control. Standard chemotherapeutic CDDP treatment (5 mg/ kg) was intraperitoneal injected each 6 days. **(A)** Tumor volume changes induced by different treatments. **(B)** Immunohistochemical staining of tumor sections to CDC47 monoclonal antibody. **(C)** Barplot representation of quantified immunohistochemical CDC47 positive staining cells in the different experimental setups (mean ± standard error of the mean (SEM) of three independent experiments). Statistical significant differences have been determined by two-way ANOVA * *p* < 0.05; ** *p* < 0.01; *** *p* < 0.001.

The CT26 syngeneic colorectal cancer tumor model was used to investigate the effect of MGF on tumor growth *in vivo.* CT26. WT cells were grown for 14 days after subcutaneous injection. Changes in tumor volume by MGF treatment were measured during 2 weeks of treatment protocol. As shown in Figure 1A, allgrafts treated with MGF show dose dependent tumor regression, comparable to the volume reduction observed upon treatment with the reference chemotherapeutic drug CDDP. Significant treatment antitumor effects start to be observed from day 22, following 8 days of treatment (Figure 1A), reaching a maximal reduction in tumor size of 75% at 100 mg/kg. Overall, CDDP treatment revealed the strongest antitumor effect (approximately 90% tumor size reduction). Along the same line, MGF and CDDP dependent reduction of tumor volume corresponds with decreased immunohistochemical staining intensity of the cell proliferation marker CDC47 reflecting arrest of tumor growth (Figures 1B,C).

### Mangiferin Inhibition of Tumor Cell Motility (Invasion), Angiogenesis, Vasculogenesis Involves Transcriptional Changes in Fatty Acid Metabolism, PPAR, NFκB, Wnt and GP6 Signaling Pathways

To get more insight in the molecular mechanism of the antitumor of MGF, a gene expression microarray experiment was performed. Gene expression profiles of subcutaneous tumor tissue obtained from mice treated for 2 weeks with 5 mg/ kg CDDP, 10 mg/ kg MGF, 50 mg/ kg MGF and 100 mg /kg were compared to untreated control mice with tumors. In general, MGF showed mild transcriptional changes as compared to CDDP treatment. Using a fold change difference of at least 1.25 and a p-value below 0.05, 454 genes were found to be differentially expressed in the 10 mg/ kg MGF condition, 263 genes in the 50 mg/ kg MGF condition, 812 genes in the 100 mg/ kg MGF condition and 3,340 genes after 5 mg/ kg CDDP treatment (Supplementary Table S1). Highly significant positive correlations could be observed upon comparing the log2 microarray gene expression values and the ΔCt qPCR expression values of *CCL3, COL6A3, CXCL9* and *MMP13* target genes, supporting the validity our microarray results (Supplementary Figure S1).

**FIGURE 2:**
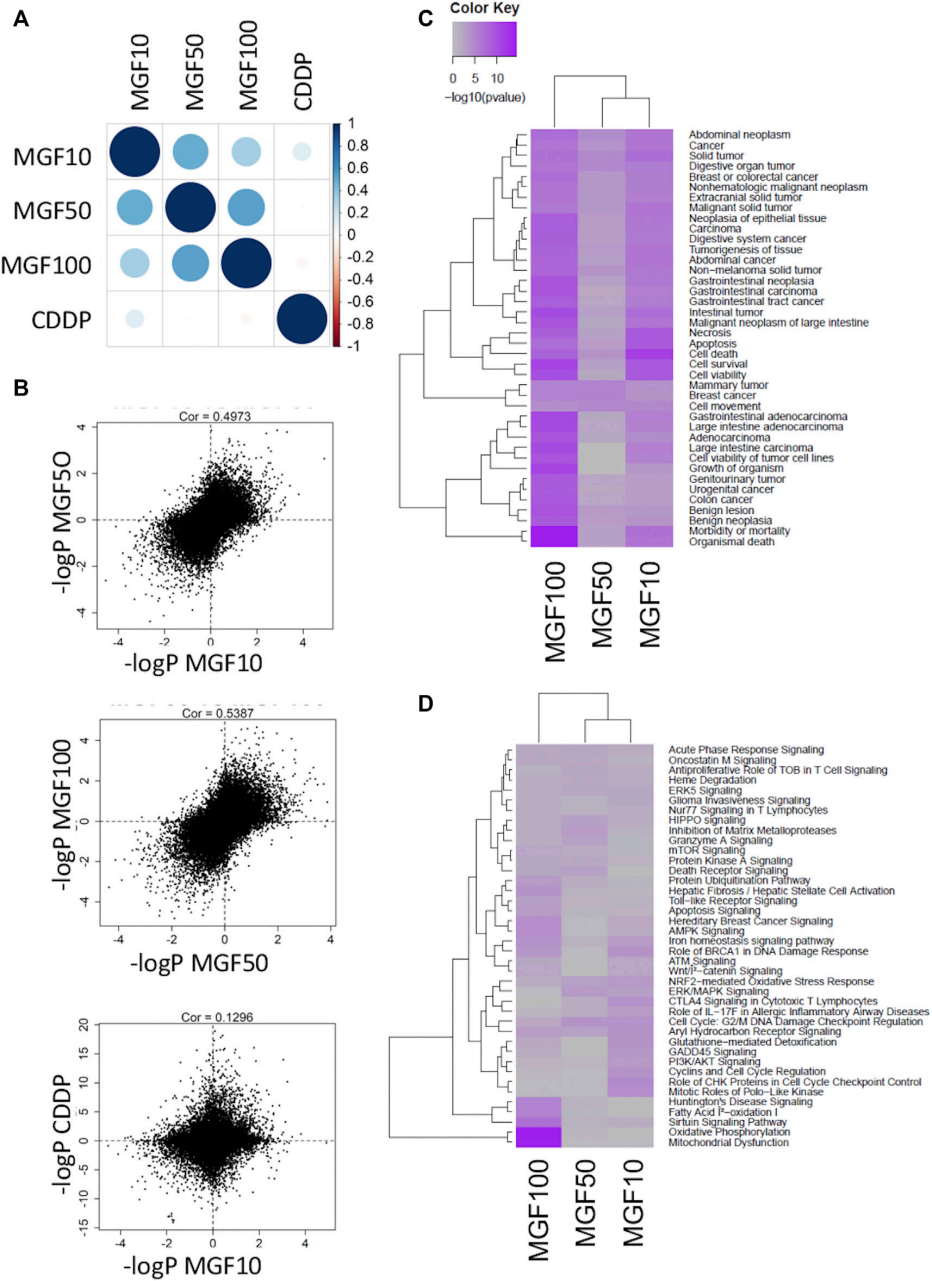
MGF treatment of CT26. WT colorectal cancer model reshapes gene expression patterns controlling cell death, cell growth, cell motility and mitochondrial disease functions. **(A)** Cross-comparison of common transcriptional changes of the different treatments by correlating the -log10 p-values for every treatment combination. **(B)** Scatter plot pearson correlations of transcriptional gene responses for different MGF doses and for MGF versus CDDP treatments **(C)** IPA pathway enrichment analysis of disease functions. **(D)** IPA pathway enrichment analysis of biological pathways.

Next, bioinformatic analysis of transcriptome changes of the different treatments was performed by correlating the -log10 p-values for every treatment combination (Figure 2A). Whereas strong positive correlations were observed for transcriptional responses for the different MGF doses (r = ≥ 0.5), only weak overlap could be observed for MGF and CDDP treatments (r = 0.13), suggesting a different mechanism of action (Figure 2B). Next, we focused on pathway enrichment analysis of disease and biological pathways of the MGF treatment specific transcriptional changes using Ingenuity Pathway Analysis (IPA^®^, QIAGEN Redwood City) (Figures 2C,D; Supplementary Table S1). Differentially expressed genes for all MGF doses are enriched for regulation of cancer and colon cancer related disease functions, including cell death and survival and cell motility, gastrointestinal neoplasia, gastrointestinal carcinoma, malignant neoplasm of large intestine, and digestive system cancer (Figure 2C).

Along the same line, significant transcriptional changes could be observed at the gene expression level in cytokine signaling (acute phase response, oncostatinM), mitochondrial metabolic stress (oxidative phosphorylation, fatty acid oxidation, Nrf2, Glutathione detoxification, SIRT, and AMPK/mTOR signaling), cell cycle (cyclins, checkpoints, mitotic PLK kinases), metalloproteases (MMPs), cell death (death receptors), iron homeostasis (heme degradation) DNA damage (ATM, BRCA1, GADD45 signaling), cell death (ubiquitination), differentiation (Wnt/catenin) and xenobiotic-metabolizing hormone receptor signaling (Aryl hydrocarbon and aldosterone receptor signaling) (Figure 2D; Supplementary Figure S3; Supplementary Table S1).

**FIGURE 3:**
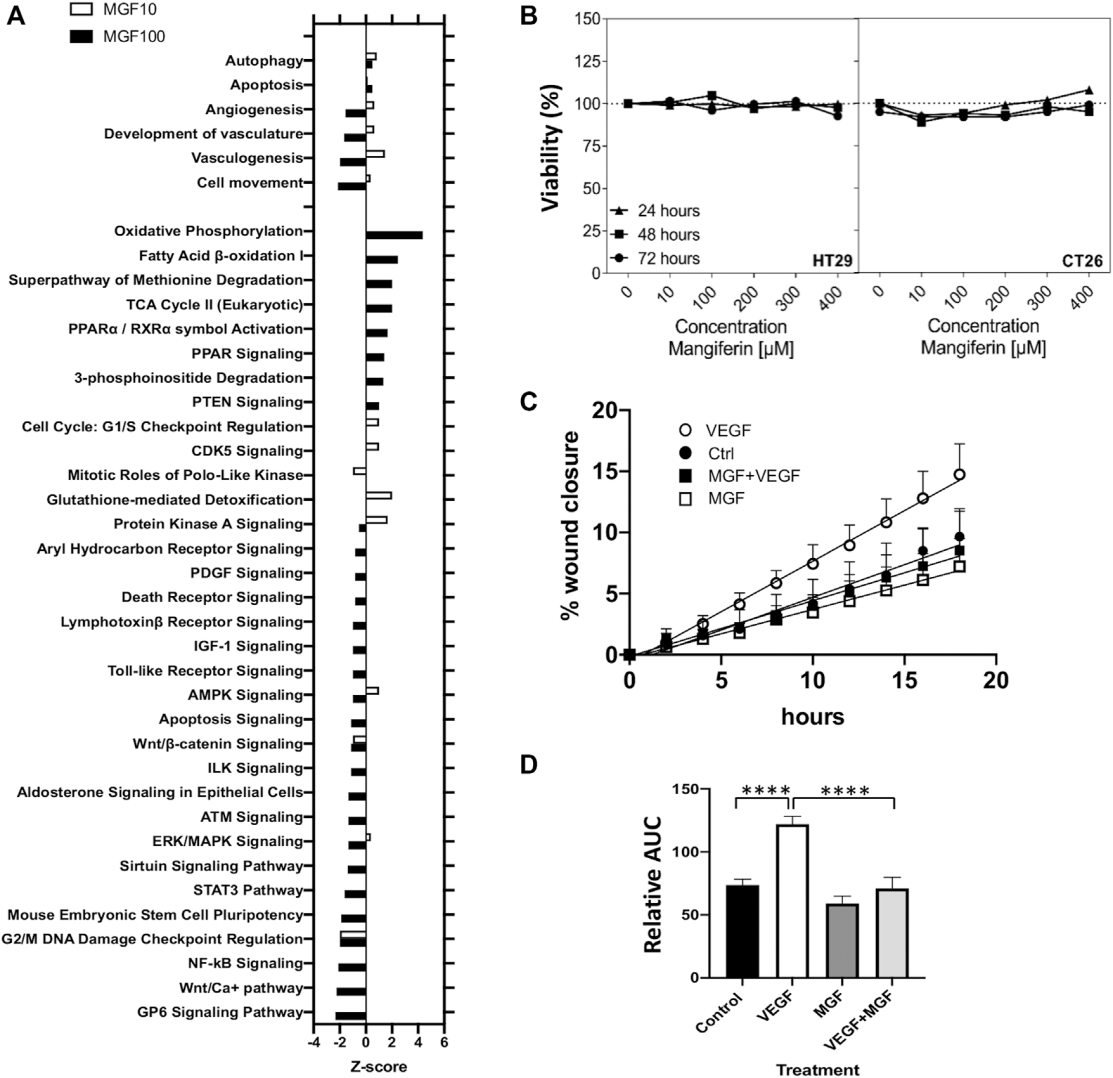
MGF treatment inhibits prometastatic tumor cell motility in a wound scratch assay *in vitro* without adverse toxicity.**(A)** Z-score barplot of most significantly enriched disease functions and canonical pathways **(B)** Assessment of cell viability by MTT assay of HT29 cells and CT26. WT colorectal cells after 24, 48 and 72 h treatment with MGF. **(C)** Realtime image quantification (0–18 h) of *in vitro* migration and wound closure of HT29 cells in the presence of 10 ng/ ml VEGF, 400 µM MGF a combination thereof or solvent matched controls. **(D)** Bar plot quantitation of the wound closure after cell scratching following different treatments by measuring each area under the curve in Figure 3C (0–18 h). Data are given as mean ± SD (*n* = 3). Statistics were calculated with 1-way ANOVA ***p < 0.0001.

Next, we used the IPA regulation z-score algorithm to identify biological functions and pathways that are most significantly activated (Z-score >1) or repressed (Z-score < -1) by MGF based on direction of gene expression changes (increase/decrease). Interestingly, MGF dose dependently suppresses expression of multiple genes involved in tumor cell motility (invasion), angiogenesis, vasculogenesis and development of vasculature functions (Figure 3A). Furthermore, when analyzing possible protein interaction networks of MGF responsive gene targets by the STRING algorithm (https://string-db.org/), we identified various interconnected protein network clusters involved in cell motility, angiogenesis and cell death/autophagy/proliferation, or dysfunctional mitochondrial oxidoreductase metabolism, in line with the results of our IPA analysis (Figures 2D, 3A and Supplementary Figure S2).

### Mangiferin Suppresses Cell Motility *in vitro*

To further characterize MGF anticancer effects on cell viability and cell motility *in vitro*, we next performed MTT and wound scratch assays in mouse CT26 and human HT29 colon cancer *in vitro* cell models. First, CT26 and HT29 were treated with MGF for 24, 48, and 72 h with a MGF concentration range from 10 to 400 μM, followed by a colorimetric MTT assay, as previously described (Hernandez-Balmaseda et al., 2021). As can be observed from Figure 3B, MGF does not significantly decrease cell viability in the dose range tested and shows no adverse toxicity. Next, MGF effects on prometastic and proangiogenic cell motility were analyzed for 24 h in a wound scratch assay in VEGF treated HT29 cells by realtime Incucyte Live Cell imaging in IncuCyte^®^ ImageLock 96-well microplates, after scratching cells with the Incucyte Woundmaker tool (Kobelt et al., 2021). Time dependent changes in wound closure of the different treatments were quantified by the Incucyte Scratch Wound Cell Migration software module of the ZOOM imaging system. In line with our gene expression pathway analysis in mouse CT26 cells, MGF treatment was also found to significantly suppress VEGF induced prometastatic and proangiogenic cell motility in human HT29 cells (Figures 3C,D).

### Mangiferin Exerts Antiangiogenic Effects *in vivo* in a Matrigel Plug Angiogenesis Mouse Model

Next, we further verified whether inhibition of vasculogenesis by MGF could also be experimentally confirmed in a CT26. WT cells engrafted matrigel plug assay, allowing to measure effects on neovascularization of tumor cells *in vivo* (Kastana et al., 2019). Angiogenesis is an attractive cancer therapeutic target, not only because it supplies oxygen and nutrients for the survival of tumor cells, but also provides the route for metastatic spread of these cancer cells.

**FIGURE 4:**
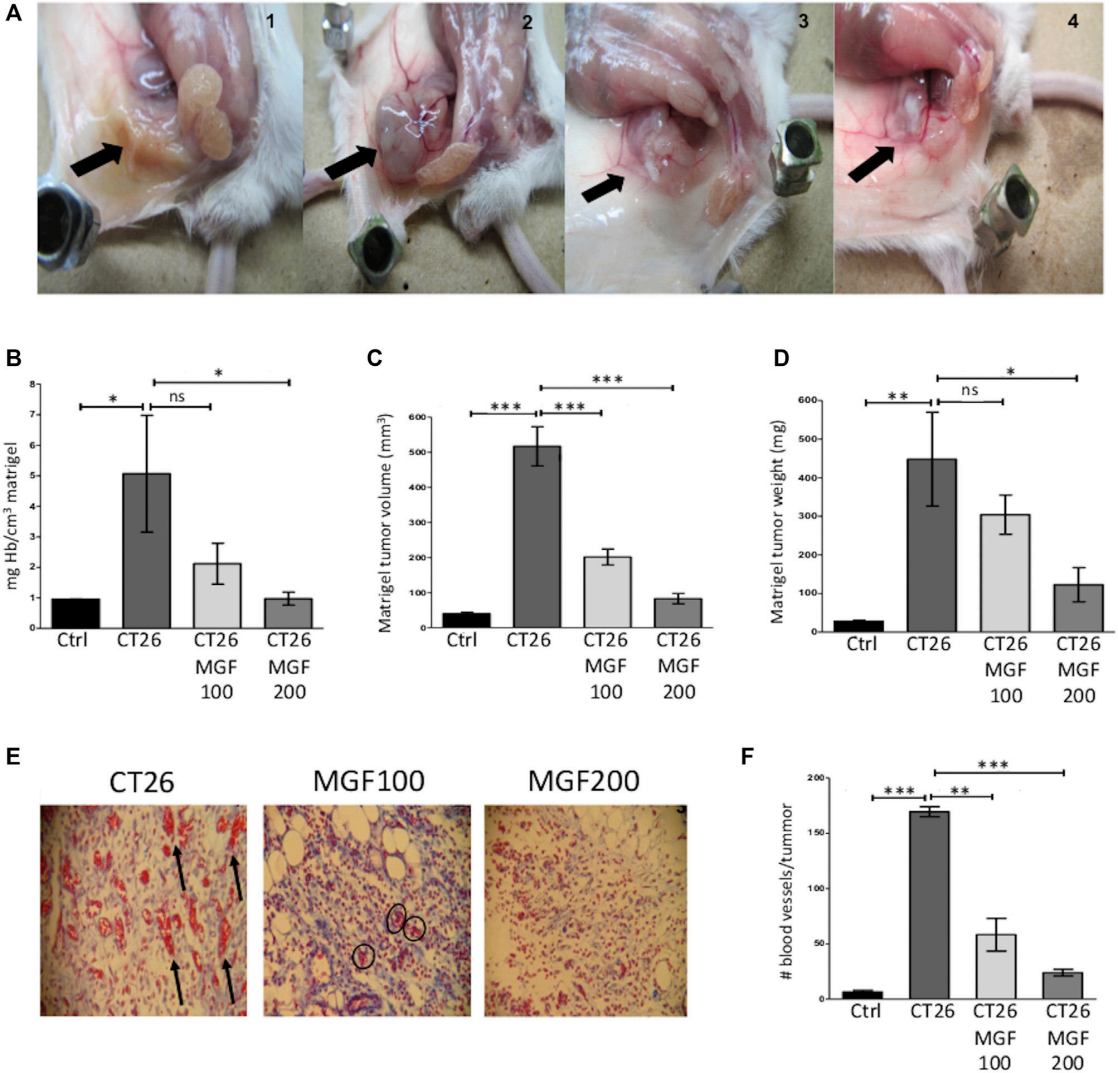
MGF treatment has antiangiogenic effects *in vivo* CT26. WT matrigel plug angiogenesis model. BALB/c mice were subcutaneously implanted bith a *basal control* plug (Matrigel + heparin + PBS), CT26 tumor plug (basal control plug + CT26. WT cells (5 x 10^5^). Treatment groups were implanted with a MGF200 plug (MGF 200 μg/ ml + Matrigel, heparin, CT26. WT, PBS) or MGF100 plug (MGF 100 μg/ ml + Matrigel, heparin, CT26. WT, PBS). Twelve days post-implantation, the subcutaneous matrigel plugs were weighed, measured, and the corresponding volumes were calculated. Hb release of liquified plugs was quantified by the Drabkin method. The histological analysis were staining by haematoxylin-eosin and subsequently with Masson’s trichrome dye. A microscopic count of the blood vessels was performed throughout the periphery of the tumours. **(A)** Macroscopic subcutaneous plug images from left to right basal control, CT26 tumor control, MGF 100 μg/ ml treatment, MGF 200 μg/ ml treatment **(B)** quantification of Hb content plug relative to tumor control plug (arbitrarily set at 100%) **(C)** volume of Matrigel plug (mm^3^) **(D)** weight of Matrigel plug (mg) **(E)** staining with Masson’s Trichrome of tumour samples. Left picture, tumour positive control plug shows numerous macro- and microvessels in the tumour periphery area, the arrow points are indicative of larger calibre blood vessels; middle picture, tumor plug with MGF100 μg/ mL shows few remaning small vessels in the matrigel (circle marks); right picture, in tumor plug with MGF 200 μg/ ml no blood vessels can be observed in the matrigel, magnification ×100. **(F)** Quantification of the number of blood vessels in the entire tumour periphery area. Data are represented as mean ± SD; not significant (ns), significant, * *p* < 0.05; *** *p* < 0.001.

Tumor formation in the subcutaneous matrigel plug tissue, with abundant blood supply, was observed in the CT26. WT engrafted control animals. In contrast, the plug in control animals colors yellow lacking blood irrigation in the matrigel subcutaneous implant (Figure 4A). Clearly, MGF treatment promotes a reduction of peritumoral and intratumoral blood vessel supply and tumor size in a dose-dependent manner (Figure 4A from left to right). Moreover, quantitative analysis of Hb content shows that MGF dose dependently inhibits angiogenesis, i.e. 58% and 81% decrease of Hb levels at respectively 100 and 200 μg/ml MGF treatment (*p* < 0.05) (Figure 4B). As expected, CT26. WT tumor cells show an adequate proangiogenic response in matrigel (81% increase in Hb content) as compared to the control setup (Figure 4B). Besides, the antiangiogenic effect of MGF also results in dose dependent reduction in tumor volume (84% reduction at 200 μg/ ml MGF, Figure 4C) or tumor weight (73% reduction at 200 μg/ ml MGF, Figure 4D). Besides, a histological study was performed using the hematoxylin-eosin and Masson’s trichrome staining. This allows clear differentiation of the collagen fibers (colored in blue) from the blood vessels (colored in red). As can be observed in the histological staining (Figure 4E) or blood vessel count (Figure 4F), MGF dose dependently inhibits the formation of blood vessels in the tumor periphery of the CT26. WT engrafted Matrigel (*p* < 0.001). Altogether, these results support our gene expression pathway enrichment analysis which identified various MGF responsive target genes involved in suppression of angiogenesis-vasculogenesis.

### Mangiferin Inhibits Lung Metastasis of CT26 Cells in an Experimental Metastasis Mouse Model

**FIGURE 5:**
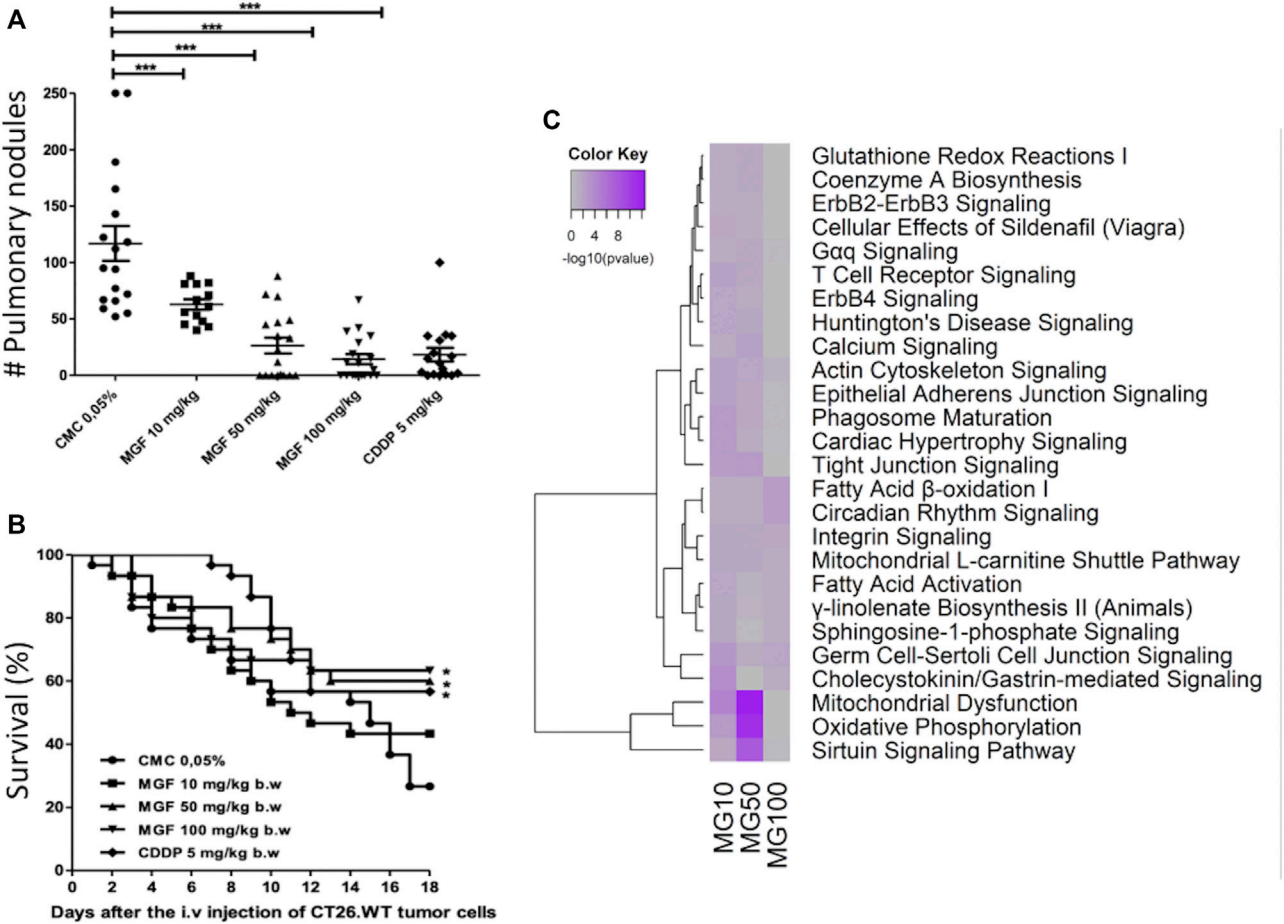
MGF inhibits CT26. WT-induced experimental lung metastasis. BALB/c mice (15/group) were orally treated with 0.05% CMC, MGF 10 mg/ kg b. w., 50 mg/ kg b. w., and 100 mg/ kg b. w. for 17 days, or intraperitoneally with 5 mg/ kg b. w of CDDP (three cycles every days), after injection of CT26. WT cells (1x10^5^ cells/100 μL non-supplemented RPMI culture medium). **(A)** number of pulmonary surface nodules counted by experimental group. **(B)** survival curves during 18 days (sacrifice) of the experiment to each experimental group. Values are shown as mean ± standard error of the mean (SEM) of three independent experiments. MGF significantly inhibited (*** *p* < 0.001) tumor lung metastasis as shown in panel A and significantly increased survival of mice as shown in panel B, as compared to vehicle control treatment response. **(C)** heatmap representations of most significantly enriched pathways associated with gene expression changes in response to MGF treatment.

To further evaluate suppression of cancer cell motility-migration in response to MGF, as predicted in our transcriptome analysis, we next investigated whether MGF could reduce number of lung metastasis nodules upon intravenous puncture of colorectal cells in the mouse tail veins. The number of CT26 metastasis nodules in lungs were quantified following 17 days treatment in the different experimental setups as shown in Figure 5. A significant reduction (*p* < 0.001) in the number of pulmonary surface metastatic nodules could be observed by increasing doses of MGF, respectively 46, 77, and 88% decrease upon treatment with 10, 50 or 100 mg/ kg b. w. MGF as compared to the vehicle control group (Figure 5A). Similarly, metastatic nodules could be reduced 83% upon treatment with 5 mg/kg b. w. CDDP (83%) (Figure 5A). Treatments with 50 and 100 mg/ kg b. w. MGF, or 5 mg/ kg b. w. CDDP also resulted in a significant improvement of longterm survival rate as compared to the vehicle control group (MGF 50 mg/ kg: *p* = 0.027; MGF 100 mg/ kg: *p* = 0.020; CDDP: *p* = 0.022) (Figure 5B). Remarkably, gene expression analysis of pulmonary metastatic colon tumor tissue biopsies from mice treated with MGF revealed similar gene expression patterns and pathway enrichment as in the primary CT26 tumor model (Figure 5C; Supplementary Table S2). Accordingly, MGF treatment targets mitochondrial energy metabolism (fatty acid oxidation, sirtuin, TCA) NFκB, PPAR, Wnt and GP6 signaling to suppress cancer cell migration-metastasis processes (Figure 5C). Since tumor tissue biopsies are a heterogenous mixture of CT26 tumor metastasis and healthy lung cells, tumor gene expression changes by MGF treatment at 100 mg/kg are less pronounced than at 50 mg/kg since the fraction of CT26 tumor cells is strongly decreased at the highest MGF concentration, which significantly reduces the MGF effect size in the mixed cell population.

### Mangiferin Regulated Protein Interaction Networks Target Mitochondrial Energy Metabolism and Inflammatory Signaling to Elicit Antitumor and Antimetastatic Therapy Response *in vivo*

**FIGURE 6:**
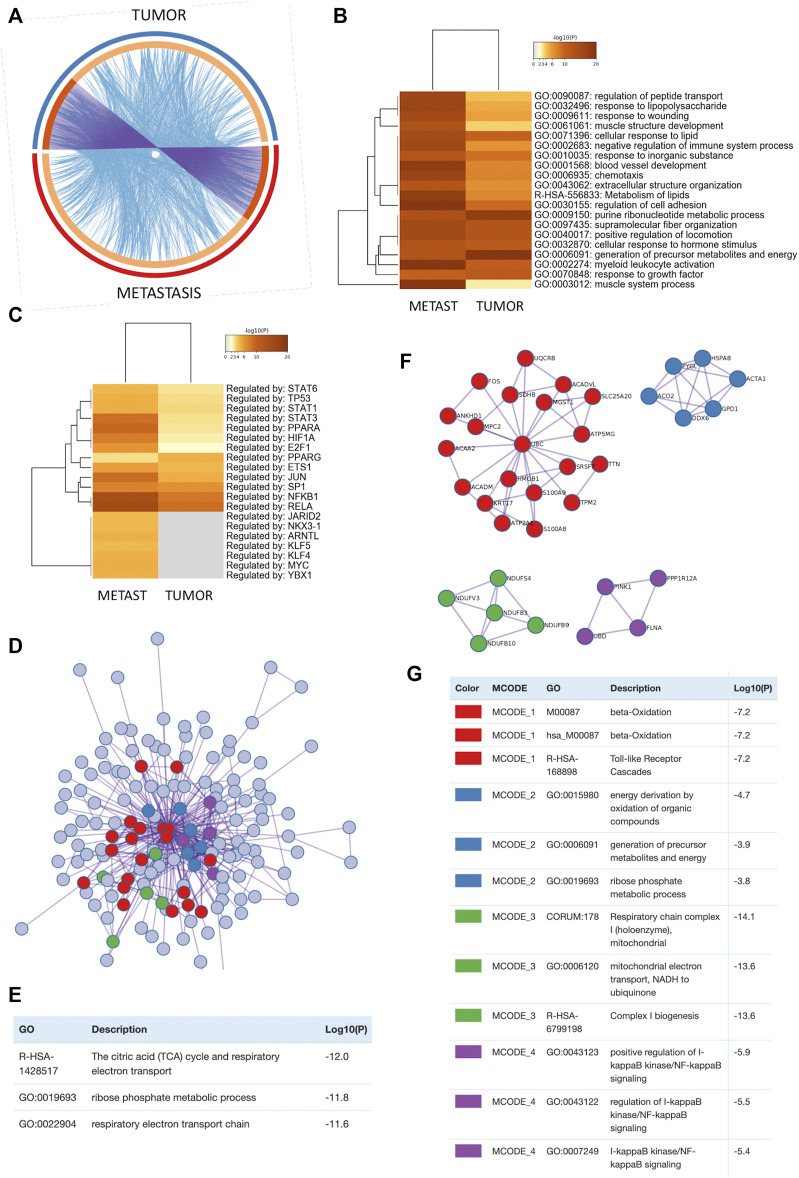
Analysis of MGF regulated protein interaction network in colorectal cancer reveals key roles for mitochondrial energy metabolism and inflammatory signaling to mediate its anticancer effects **(A)** The Circos plot shows how genes from the different input gene lists of tumor (TUMOR) *vs*. metastasis (METAST) mouse models overlap. On the outside, arc represents the identity of each gene list. On the inside, orange color represents the gene names that appear in both lists and light orange color represent unique gene names that are part of common enriched pathways (same ontology term). Purple lines link the same gene shared by multiple gene lists. Blue lines link the genes where they fall into the same ontology term (the term has to be statistically significantly enriched). The greater the number of purple links and the longer the dark orange arcs implies greater overlap among the input gene lists. Blue links indicate the amount of functional overlap among the input gene lists. **(B)** Metascape enrichment analysis of all statistically enriched ontology terms (GO/KEGG terms, canonical pathways, hall mark gene sets). **(C)** Metascape enrichment analysis of all statistically enriched TF-target interaction networks. **(D)** All protein-protein interactions (PPI) among all input gene lists were extracted from PPI data source and formed a PPI network. **(E)** GO enrichment analysis was applied to assign biological “meanings” of the PPI network. **(F)** MCODE sub-protein network components were identified from the merged PPI network. Each MCODE network is assigned a unique color. **(G)** GO enrichment analysis was applied to assign biological “meanings” of MCODE sub-protein networks, where top three best p-value terms were retained.

To further characterize molecular mechanism of action of MGF, we further crosscompared MGF responsive genes in the primary CT26 tumor model and the metastatic cancer models by Metascape systems biology freeware (https://metascape.org/) (Zhou et al., 2019) (Supplementary Figure S4; Supplementary Table S3). As can be observed in the circos plot (Figure 6A), we observe significant overlap between gene expression changes as well as biological pathways, as both experiments probably target similar biological processes. This is also clear in the overlapping heatmap representation (Figure 6B), showing highly significant pathway enrichment related to blood vessel development, lipid metabolism, chemotaxis, locomotion, wound healing, generation of energy metabolites, hormone stimulus, growth factor response and leukocyte activation among others (Supplementary Table S3). Further TRRUST analysis of all statistically enriched TF-target interaction networks (Koundouros and Poulogiannis, 2020) identified major regulatory roles for STAT3, PPARA/G, HIF1A and NFκB1/RelA transcription factor families, which are key players in lipid metabolism, hypoxia and inflammatory tumor signaling (Figure 6C). Furthermore, Metascape protein-protein-interaction (PPI) analysis of all MGF responsive target genes reveals a global protein network involved in reprogramming of mitochondrial energy metabolism in CT26 tumor cells (Figures 6D,E). More specifically, 4 major protein interaction subnetworks could be identified (Figure 6F) involved in fatty acid β-oxidation and toll like receptor signaling (MCODE1), generation of energy metabolites (oxidation organic compounds, ribose phosphate metabolism) (MCODE2), mitochondrial respiratory chain complex 1 (MCODE3) and NFκB signaling (MCODE4) (Figure 6G).

### Mangiferin Targets Fatty Acid β-oxidation Metabolism in Human HT29 Colon Cancer Cells

**FIGURE 7:**
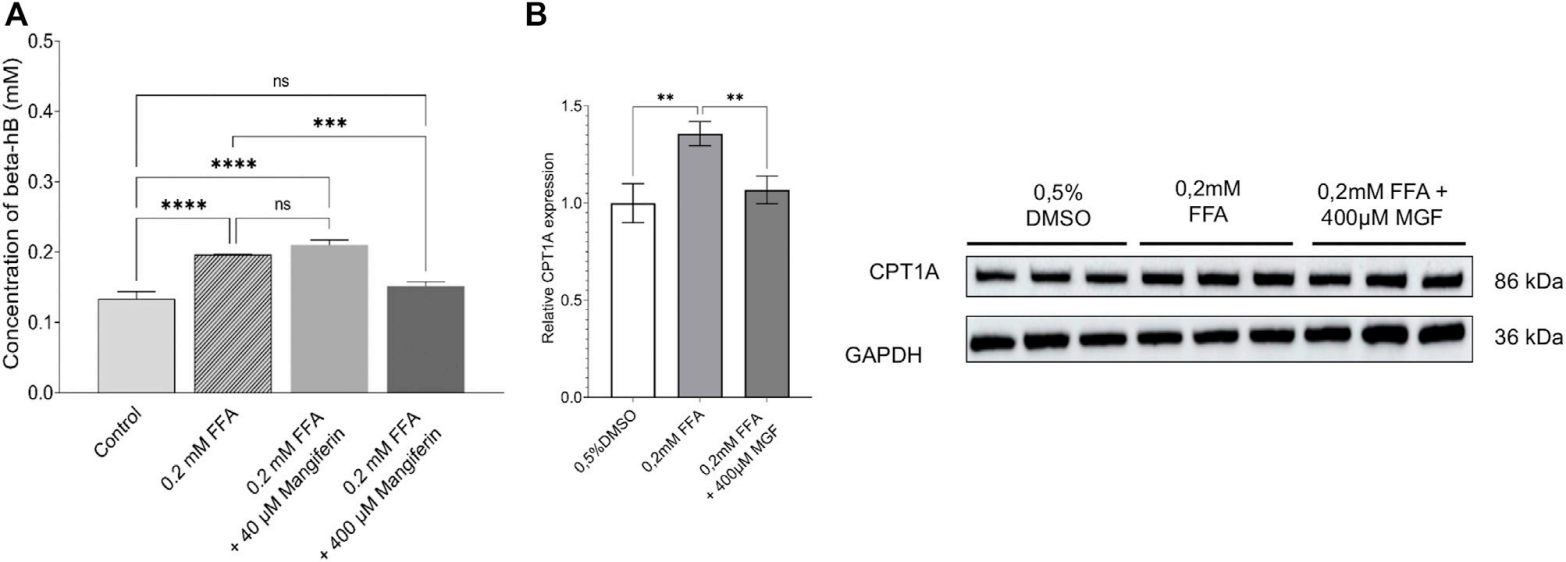
MGF decreases free fatty acid induced fatty acid β-oxidation metabolism in human HT29 colon cancer cells. **(A)** Intracellular concentrations of β-hydroxybutyrate in HT29 cells in response to 24 h treatment with 0.2 mM free fatty acids (FFA) increase significantly compared to untreated HT29 cells. Cotreatment with 400 µM MGF reverses this effect. Data are given as mean ± SD (*n* = 3 wells). Statistics were calculated with 1-way ANOVA and Tukey post-test; ***p < 0.001; ****p < 0.0001; ns indicates not significant. **(B)** Representative quantification **(left)** of western blot analysis of relative CPT1A protein expression **(right)** in HT29 cells after treatment with 0.2 mM FFA or 0.2 mM FFA with 400 µM MGF. The relative levels of CPT1A were quantified by normalizing to GAPDH which was used as a loading control. Data are given as mean ± SD of three biological replicates. Statistics were calculated with 1-way ANOVA and Dunnett’s adjustments; **p < 0.01 compared to FFA treated cells.

To verify whether MGF directly targets fatty acid β-oxidation energy metabolism in colon cancer cells, we measured ketone body concentrations (β-hydroxybutyrate) upon exposure of human HT29 colon cancer cells to free fatty acids (to mimick free fatty acids present in the tumor microenvironment *in vivo*) in presence or absence of MGF. Figure 7A shows that 24 h treatment of HT29 cells with 0.2 mM free fatty acids (FFA) increases intracellular concentrations of β-hydroxybutyrate as compared to untreated HT29 cells. Of special note, cotreatment with 400 µM MGF reverses this effect to levels observed in untreated cells. Along the same line we measured changes in protein expression of carnitine palmitoyltransferase (CPT1), a key metabolic enzyme involved in fatty acid β-oxidation. Similarly, FFA treatment increases CPT1 expression levels whereas, MGF reverses the effect to control levels (Figure 7B).

## Discussion

Today, various pharmacological anticancer properties of MGF have already been described *in vitro*, including antiproliferative, pro-apoptotic, anti-invasive, antimetastatic and antiangiogenic bioactivities (García-Rivera et al., 2011; Khurana et al., 2016; Núñez Selles et al., 2016; Imran et al., 2017; Delgado-Hernández et al., 2020). Here, we further demonstrate *in vivo* antitumor efficacy of MGF in different syngeneic immune competent colon cancer (CT26) mouse allograft models and propose a novel “metabolic” anti-cancer mechanism of action.

To further characterize the corresponding molecular mechanisms involved in tumor regression we have applied systems biology pathway enrichment analysis (IPA) of gene expression patterns of tumor samples of the various treatments. This revealed that cell motility, invasion and metastasis are the most significantly suppressed biological pathways upon MGF treatment. This could be confirmed human and mouse colon cancer cells in a wound scratch assay *in vitro* and a lung metastasis model *in vivo*. At the gene expression level, MGF suppresses various prometastatic genes, including MMP13, COL4A1, COL4A2, INHBA, KMT2A, ZEB1/2. Tumour invasion and metastasis involves degradation of different components of the extracellular matrix and basement membranes (Type IV collagen), catalysed by proteolytic enzymes such as matrix metalloproteinases (MMPs). Degradation of type IV collagen correlates with metastatic potential, whereas MMP-13 gene expression has been identified as a reliable predictor of liver metastasis in patients with CRC (Yamada et al., 2010).

Serum levels of Collagen IV (Col4A1, Col4A2) abundant in are elevated in patients with colorectal liver metastases and facilitate invasion (Wang et al., 2020; Liu et al., 2020). The heterodimeric TGF-β family ligand Inhibin (INHA) is a novel paracrine factor involved in cancer angiogenesis and metastasis (Singh et al., 2018a). The histone methyltransferase KMT2A promotes colorectal cancer metastasis via activation of cathepsin Z (Fang et al., 2019). Finally PDPK1 and ZEB1/2 members drive tumor metastasis spread via regulating epithelial–mesenchymal transition (EMT) during hypoxia (Dupuy et al., 2015; Du et al., 2016; Stemmler et al., 2019; Saxena et al., 2020). Since angiogenesis is one route for metastatic spread of cancer cells, it can be noticed that various transcriptional changes blocking angiogenesis, indirectly also reduce metastatic cell migration and both pathways are interconnected (Supplementary Figure S2). Accordingly, MGF decreased expression of various proangiogenic genes such as HMOX1, MMP13, IGF1R, INSR, TGFB3, PDGFR A/B and SEMA5A among others. HMOX1 promotes vascularisation in a hypoxic environment by stimulating paracrine proangiogenic functions of bone marrow derived cells (BMDCs) (Grochot-Przeczek et al., 2014). Besides, HMOX1 also promotes proangiogenic effects in endothelial cells by reciprocal regulation of VEGF and SDF1 (Ayer et al., 2016). TGFB3 induces angiogenesis by promoting transdifferentiation of mesenchymal stem cells into endothelial cells (Batlle et al., 2019). PDGFRA and PDGFRB play different roles in neosvascularisation and promoting vessel stability (Zhang et al., 2009). Matrix metalloprotein (MMP13) by degrading extracellular matrix (ECM) stimulates the release of VEGF from ECM and thereby promoting angiogenesis and metastasis in skin squamous cell carcinoma and colitis induced colon cancer (Lederle et al., 2010; Ai et al., 2015). SEMA5A promotes angiogenesis by increasing endothelial cell proliferation, migration (Sadanandam et al., 2010). Furthermore, oncogenic angiogenic and metastatic functions have been described for INS and IGF1R (Heidegger et al., 2014). Finally, in line with the bioinformatic analysis, the predicted inhibition of angiogenesis, and suppression of vasculogenesis could also be confirmed in an *in vivo* matrigel plug implant mouse model.

By crosscomparing MGF responsive genes in the primary CT26 tumor model and the metastatic cancer models by Metascape systems biology freeware (https://metascape.org/), we searched for a common molecular mechanism of action (Figure 6). Interestingly, significant enrichment of various common pathways could be observed related to blood vessel development, lipid metabolism, chemotaxis, locomotion, wound healing, generation of energy metabolites, hormone stimulus, growth factor response and leukocyte activation among others Furthermore, we identified major regulatory roles for STAT3, PPARA/G, SIRT, HIF1A and NFκB1/RelA transcription factor families, which are key players in lipid metabolism, hypoxia and inflammatory tumor signaling. Ultimately, protein-protein-interaction (PPI) analysis of all MGF responsive target genes established a global protein network with 4 major protein interaction subnetworks involved in fatty acid β-oxidation and toll like receptor signaling (MCODE1), generation of energy metabolites (oxidation organic compounds, ribose phosphate metabolism) (MCODE2), mitochondrial respiratory chain complex 1 (MCODE3) and NFκB signaling (MCODE4). MGF regulation of fatty acid β-oxidation metabolism could further be experimentally validated in human HT29 colon cancer cells by colorimetric detection of decrease in β-hydroxybutyrate concentrations and protein expression of the key metabolic enzyme CPT1. Ketone bodies are interwoven with crucial mammalian metabolic pathways such as β-oxidation (FAO), the tricarboxylic acid cycle (TCA), gluconeogenesis, glycolysis and the pentose phosphate pathway (PPP) (Puchalska and Crawford, 2017), which were identified in Metascape protein-protein interaction analysis as key protein (sub)networks affected by MGF treatment (Figure 6). A common feature of cancer cells is their ability to rewire their metabolism to sustain the production of ATP and macromolecules needed for cell growth, division and survival. These alterations are pivotal to the development and maintenance of the malignant phenotype of cancer cells in unfavorable tumor microenvironment or metastatic sites. In particular, the importance of altered fatty acid metabolism in cancer has received renewed interest as, aside their principal role as structural components of the membrane matrix, they are important secondary messengers, and can also serve as fuel sources for energy production (Koundouros and Poulogiannis, 2020; Nagarajan et al., 2021). The ways in which cancer cells utilize lipids are often influenced by the complex interactions within the tumor microenvironment and adjacent stroma (Ma et al., 2018; Corn et al., 2020). Cancer cells survive in challenging hypoxic or nutrient depleted tumor micro-environments. For example tumor-stroma localized adipocytes can be activated by cancer cells to lipolyze their triglyceride stores, delivering secreted fatty acids to cancer cells for uptake through numerous fatty acid transporters. Cancer-associated fibroblasts are also implicated in lipid secretion for cancer cell catabolism and lipid signaling leading to activation of mitogenic and migratory pathways (Koundouros and Poulogiannis, 2020; Nagarajan et al., 2021). As these cancer-stromal interactions are exacerbated during tumor progression, fatty acids secreted into the microenvironment can impact infiltrating immune cell function and phenotype. Regulation of TLR/NFκB/PPAR pathways by (un)saturated fatty acids plays a key role in tumor-immune responses (Hwang et al., 2016). Lipid metabolic abnormalities such as increased fatty acid oxidation and *de novo* lipid synthesis can provide survival advantages for the tumor to resist chemotherapeutic and radiation treatments and alleviate cellular stresses involved in the metastatic cascade. Fatty acid oxidation is also reprogrammed in cancer-associated immune and other host cells, which may contribute to immune suppression and tumorpromoting microenvironment. New evidence indicates that cancer stem cells show high metabolic plasticity and can dynamically transform their metabolic state to favor glycolysis or oxidative metabolism depending on the tumor microenvironment (Belisario et al., 2020; Zhu et al., 2020). This illustrates that fatty acids regulate each part of the cancer lifecycle and suggests that therapeutic intervention targeting lipid and fatty acid metabolism signaling pathways by MGF holds promise for colorectal cancer treatment (Kadochi et al., 2017; Nakamura et al., 2018; Li et al., 2021). Fatty acid catabolism and anabolism pathway crosstalk are pivotal in cell fate decision during redox regulated ferroptosis cancer therapy and maybe strongly context dependent (Hassannia et al., 2018; Kumar et al., 2021; Yuan et al., 2021). In clinical trials, MGF treatment was already found to improve metabolic health (diabetes, hyperlipidemia, insulin resistance) by optimization of mitochondrial bioenergetic pathways (fuel switching between fatty acid and carbohydrates) *via* sirtuin (SIRT) and PPAR (in)dependent metabolic mechanisms (Guo et al., 2011; Niu et al., 2012; Apontes et al., 2014; Na et al., 2015; Singh et al., 2018b; Li et al., 2018; Liu et al., 2018; Zhang et al., 2019). Sirtuin NAD + -dependent histone deacetylase enzymes are central players in the maintenance of cellular energy and metabolic homeostasis, whereas impaired SIRT functions result in several pathological conditions and contribute to the altered metabolic phenotype (metastasis, drug resistance) of malignantly transformed (colorectal) cancer cells, referred to as the Warburg effect (Chalkiadaki and Guarente, 2015; Gaál and Csernoch, 2020). Natural product SIRT modulators (such as MGF) are receiving growing interest to optimize mitochondrial bioenergetic pathways in cancer treatment (Iside et al., 2020; Karaman Mayack et al., 2020). Similarly, PPAR transcription factors are fatty acid sensors that enhance mitochondrial fatty acid β-oxidation (Poulsen et al., 2012; Puchalska and Crawford, 2017). Besides their lipid metabolic effects, PPARs also suppress angiogenesis, metastasis by targeting HIF, NFκB and STAT3 TF functions which regulate cancer hypoxia, inflammatory pathways, chemotaxis, cell adhesion (tight junctions), epithelial-mesenchymal transition (wnt signaling) in the tumor-microenvironment ((Michalik et al., 2004; Wang et al., 2004; Panigrahy et al., 2005; Moraes et al., 2010; Zhou et al., 2012; Woolf et al., 2015; Gou et al., 2017; Ju et al., 2017; Vu and Datta, 2017; Vallée and Lecarpentier, 2018; Mammadova-Bach et al., 2020), in line with our systems biology network analysis. Today, various natural and pharmacological PPAR ligands have already been described with anti-metastatic and anti-angiogenic therapeutic properties (Panigrahy et al., 2005; Gou et al., 2017), which increase the efficacy of chemotherapeutics with minimal toxicity (Girnun et al., 2007; Girnun et al., 2008). Similarly, our results further suggest that MGF has potential value to be used as adjuvant phytotherapeutic treatment against CRC to reduce adverse toxicity and to mitigate outcome of colon cancer treatment. To further develop its application in clinical practice, more molecular combination studies with different MGF formulations (du Plessis-Stoman et al., 2011; Morozkina et al., 2021; Baán et al., 2019) as well as uniformly controlled clinical trials are needed.

## Data Availability

All transcriptome data have now been deposited in a public depository (https://zenodo.org/) and can be accessed *via* https://doi.org/10.5281/zenodo.5185362.
