# Supplementary figures and images for: Antiproliferative, Antiangiogenic, and Antimetastatic Therapy Response by Mangiferin in a Syngeneic Immunocompetent Colorectal Cancer Mouse Model Involves Changes in Mitochondrial Energy Metabolism
Source: Front Pharmacol. 2021 Dec 3;12:670167. doi: 10.3389/fphar.2021.670167 (PMC8678272; doi:10.3389/fphar.2021.670167)

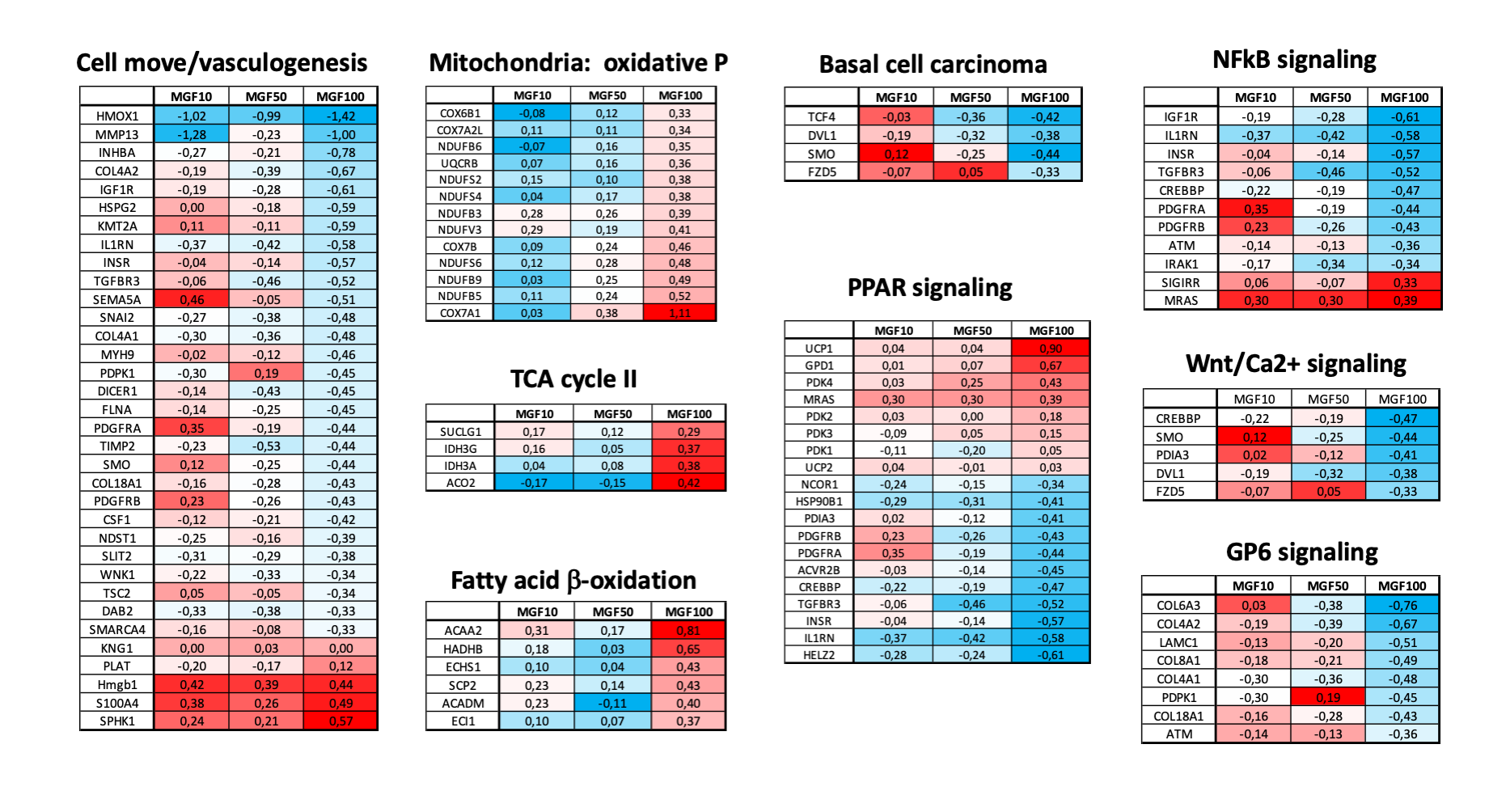

Supplement: Supplementary file 1 [file Image3.TIFF]

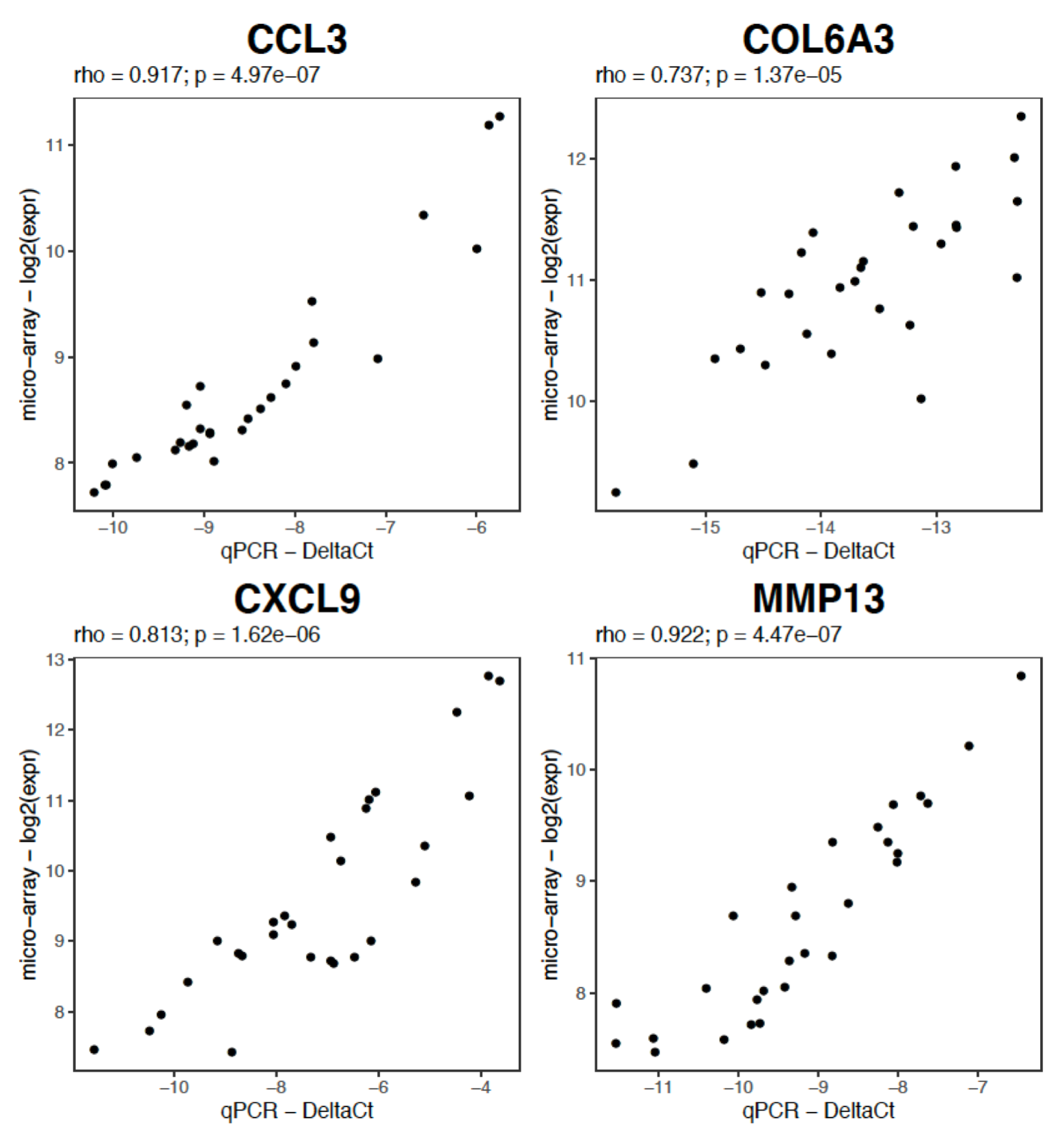

Supplement: Supplementary file 3 [file Image1.TIFF]

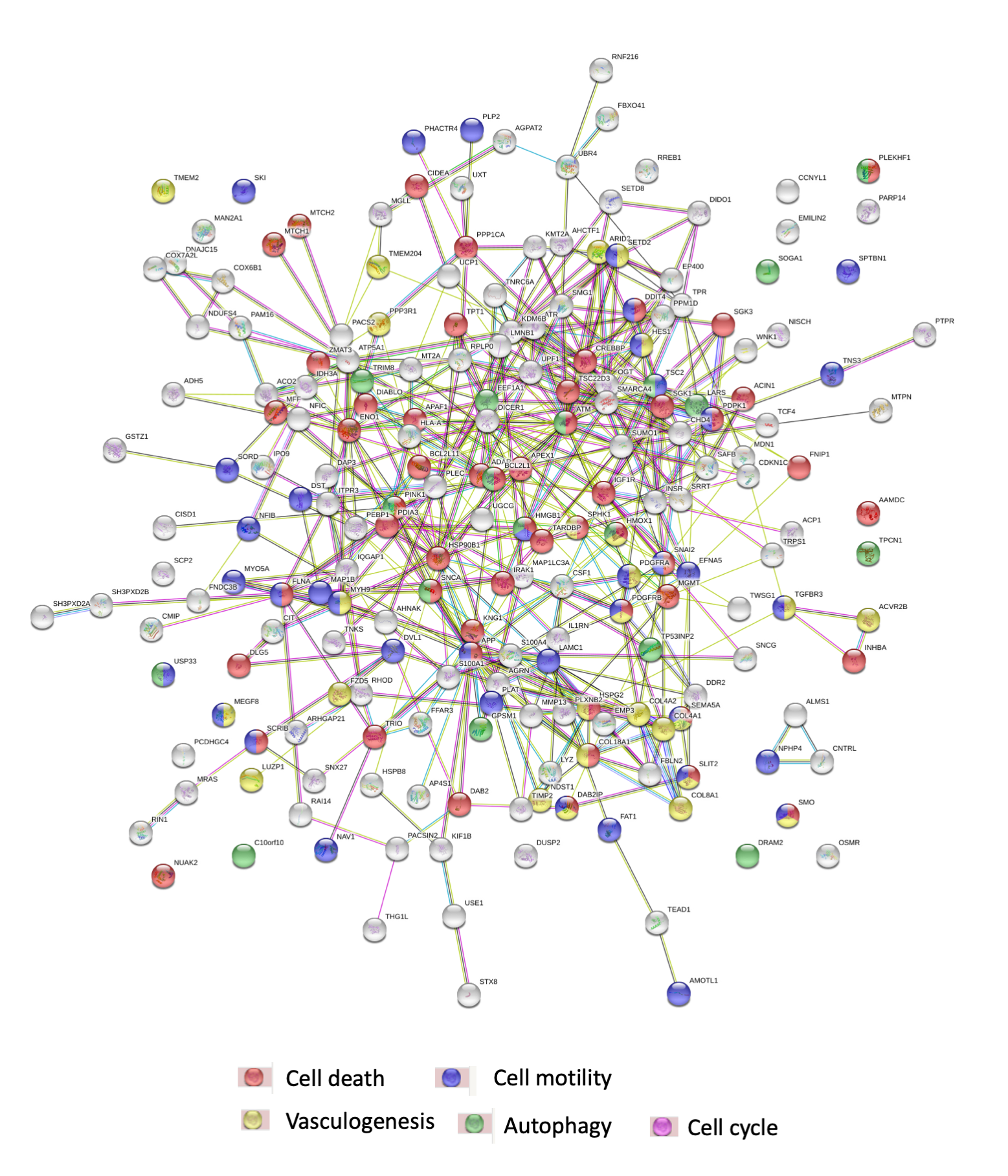

Supplement: Supplementary file 6 [file Image2.TIFF]

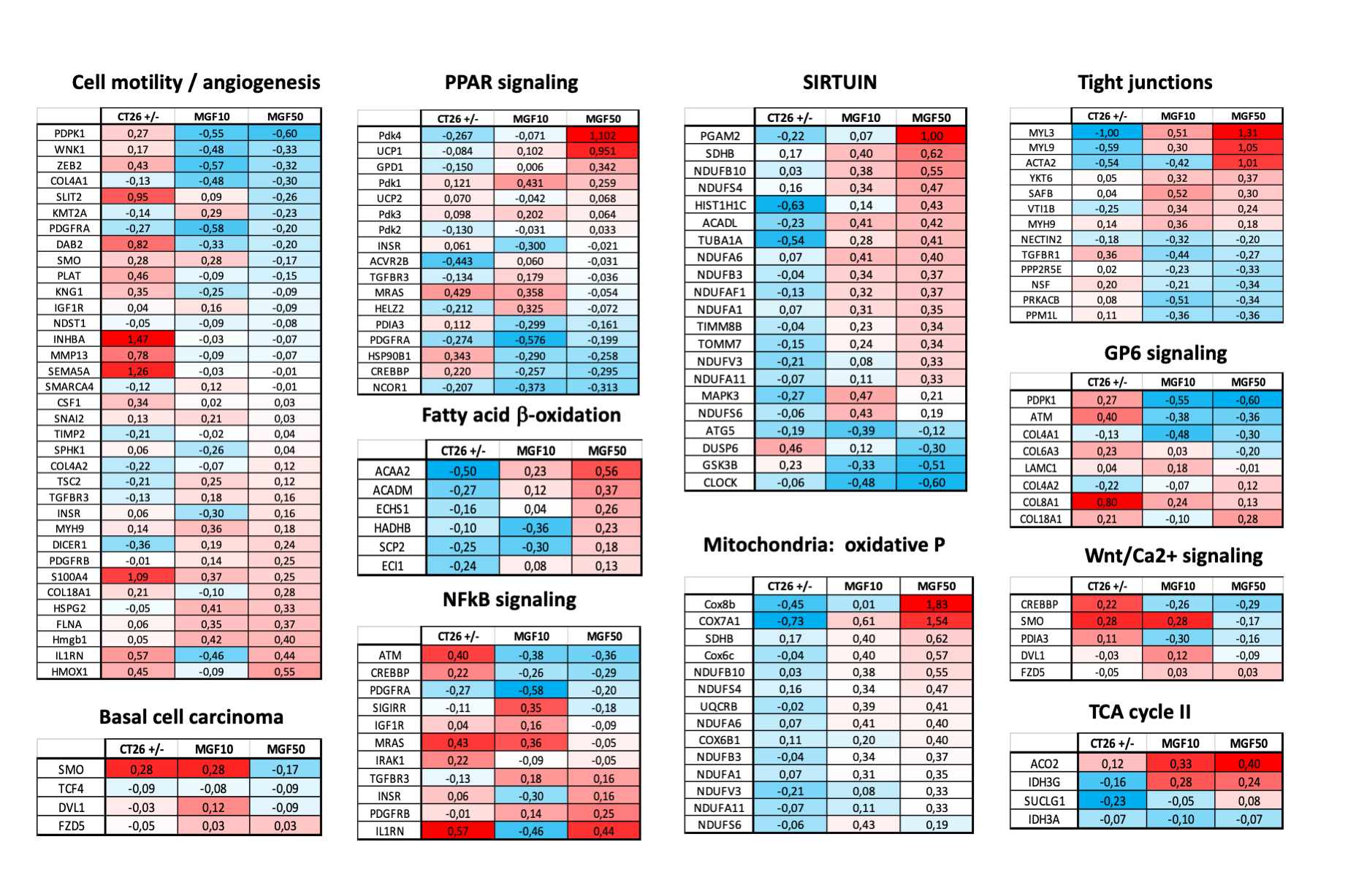

Supplement: Supplementary file 7 [file Image4.TIFF]
